# Fecal Microbial Composition of Ulcerative Colitis and Crohn’s Disease Patients in Remission and Subsequent Exacerbation

**DOI:** 10.1371/journal.pone.0090981

**Published:** 2014-03-07

**Authors:** Edgar S. Wills, Daisy M. A. E. Jonkers, Paul H. Savelkoul, Ad A. Masclee, Marieke J. Pierik, John Penders

**Affiliations:** 1 School for Nutrition, Toxicology and Metabolism (NUTRIM), Division Gastroenterology-Hepatology, Maastricht University Medical Center+, Maastricht, The Netherlands; 2 School for Nutrition, Toxicology and Metabolism (NUTRIM), Department of Medical Microbiology, Maastricht University Medical Center+, Maastricht, The Netherlands; 3 School for Public Health and Primary Care (Caphri), Department of Epidemiology, Maastricht University, Maastricht, The Netherlands; Argonne National Laboratory, United States of America

## Abstract

**Background:**

Limited studies have examined the intestinal microbiota composition in relation to changes in disease course of IBD over time. We aimed to study prospectively the fecal microbiota in IBD patients developing an exacerbation during follow-up.

**Design:**

Fecal samples from 10 Crohn’s disease (CD) and 9 ulcerative colitis (UC) patients during remission and subsequent exacerbation were included. Active disease was determined by colonoscopy and/or fecal calprotectine levels. Exclusion criteria were pregnancy, antibiotic use, enema use and/or medication changes between consecutive samples. The microbial composition was assessed by 16S rDNA pyrosequencing.

**Results:**

After quality control, 6,194–11,030 sequences per sample were available for analysis. Patient-specific shifts in bacterial composition and diversity were observed during exacerbation compared to remission, but overarching shifts within UC or CD were not observed. Changes in the bacterial community composition between remission and exacerbation as assessed by Bray-Curtis dissimilarity, were significantly larger in CD versus UC patients (0.59 vs. 0.42, respectively; p = 0.025). Thiopurine use was found to be a significant cause of clustering as shown by Principal Coordinate Analysis and was associated with decreases in bacterial richness (Choa1 501.2 vs. 847.6 in non-users; p<0.001) and diversity (Shannon index: 5.13 vs. 6.78, respectively; p<0.01).

**Conclusion:**

Shifts in microbial composition in IBD patients with changing disease activity over time seem to be patient-specific, and are more pronounced in CD than in UC patients. Furthermore, thiopurine use was found to be associated with the microbial composition and diversity, and should be considered when studying the intestinal microbiota in relation to disease course.

## Introduction

Crohn’s disease (CD) and ulcerative colitis (UC) are chronic inflammatory diseases of the gastrointestinal tract, collectively referred to as inflammatory bowel disease (IBD). IBD is a heterogenous disease ith respect to disease location, disease course, occurrence of extra-intestinal manifestations and therapeutic response. The disease course is characterized by exacerbations and remissions. IBD is generally considered to arise from the interaction between host genetics, environmental factors, dysregulated immune responses and alterations in the intestinal microbiota composition [1]. IBD, especially active disease, is associated with a decreased quality of life and high health care costs, [2], [3] especially due to the use of medication [2], [4]. Treatment is mainly based on symptom reduction by nonspecific immune-modulating drugs and can be associated with serious side effects [5]. Further insight in causative factors associated with the development of exacerbations (i.e. active mucosal inflammation) may contribute to new specific treatment options for IBD.

The intestinal microbiota is considered to play a central role in the pathogenesis of IBD and numerous studies have corroborated evidence for intestinal dysbiosis in IBD patients compared to healthy controls [6]–[9]. The gut microbiota of healthy individuals is dominated by the bacterial phyla Firmicutes and Bacteroidetes, and to a lesser extent by Proteobacteria, Actinobacteria and Verrucomicrobia [10]. In IBD patients, members of the Firmicutes phylum appear to be reduced, [9], [11], [12] whereas members of Gammaproteobacteria seem to bloom [13]–[16]. In CD patients the Clostridia cluster IV group, in particular *Faecalibacterium prausnitzii,* has shown to be decreased [15], [17]. Members of Clostridia group XIVa, belonging to the *Roseburia* genus, also seem to be decreased in all IBD patients [17]–[19]. Data on Bacteroidetes are more ambiguous; inconsistent findings have been reported for their presence in IBD compared to controls [20]–[25]. In addition to these differences in relative abundances of specific phylotypes, there appears to be a general decrease in biodiversity in IBD patients [25], [26].

Altogether, these studies provide compelling evidence for changes in the gut microbial communities in IBD patients as compared to healthy controls. Only a few studies, however, investigated the intestinal microbiota in relation to disease activity. Differences in bacterial species or groups (e.g. *F. prausnitzii*, Clostridia, *E. coli* and *Fusobacterium varium*) were found, when comparing active with inactive UC and CD patients,[7], [9], [20], [27]–[29] but could not always be confirmed [17]. These studies are prone to confounding effects due to their cross-sectional designs and differences in medication use. Studies monitoring the microbiota composition within patients with changing disease activity over time and controlling for medication use are currently lacking. Therefore, the aim of the present study was to prospectively monitor the microbial composition in UC and CD patients during remission and subsequent exacerbation by means of 16S rDNA pyrosequencing.

## Materials and Methods

### Population and Design

The current study was conducted within the context of a prospective follow-up cohort of IBD outpatients, in which standardized demographic and clinical data, feces and blood samples were collected at study entry, at every subsequent outpatient visit and during an exacerbation [30].

The diagnosis of IBD was based on clinical and endoscopic or radiological findings. For case definition of CD, the Lennard-Jones criteria were applied [31]. UC was defined as continuous mucosal inflammation with granulomata, affecting the rectum and/or some or all of the colon in continuity with the rectum. Data of concomitant use of medication, body mass index (BMI), disease duration since diagnosis and disease phenotype (Montreal classification) were obtained using the computer-based medical registration databases. Furthermore at each visit, the occurrence of infections was checked by medical history and culture if indicated, and questions on overall changes in dietary habits were completed.

Fecal samples were collected at home on the evening before or the morning of each visit and stored at 4°C. Upon arrival at the hospital, the faecal samples were split directly. Part was sent to the laboratory of Clinical Chemistry for routine analysis of fecal calprotectine and the remaining part was frozen at −80°C within 24 hours after defecation for analyses of the microbiota.

Within the prospective cohort, patients with faecal samples at remission as well as after subsequent development of an exacerbation during a one-year follow-up period were eligible for the present study.

As clinical indices do not correlate very well with endoscopic scores [32], [33] and as we aimed to assess the association of the fecal microbiota with the presence of mucosal inflammation, an exacerbation was defined by colonoscopy, a calprotectin value >150 µg/g feces or >5-fold increase in calprotectin as compared to remission levels. For CD, only patients with colonic involvement were included as fecal samples are not likely to reflect microbial composition in the small intestine. For UC, eligible patients had pancolitis, left-sided colitis or proctosigmoiditis. Exclusion criteria were pregnancy, use of rectal enemas, use of antibiotics in the 3 months prior to inclusion or during follow-up, and/or a change in immunosuppressive medication between sampling at remission and subsequent development of disease exacerbation.

### Ethics Statement

The study was approved by the Medical Ethics Committee of the Maastricht University Medical Center+ (NL31636.068.10), and written informed consent was obtained from all subjects.

### 16S rRNA V1–V3 Amplicon Library Preparation

Approximately 200 milligram feces was used for DNA isolation by the PSP SPIN Stool DNA plus kit (Stratec Molecular GmbH, Berlin, Germany) according to the manufacturer’s instructions and eluted in a final volume of 200 µL.

Amplicon libraries for pyrosequencing of the 16S rDNA V1–V3 regions were generated using a barcoded forward primer consisting of the 454 Titanium platform A linker sequence (5′-CCATCCCTGCGTGTCTCCGACTCAG-3′), a key (barcode) that was unique for each sample, and the 16S rRNA 534R primer sequence 5′- ATTACCGCGGCTGCTGG -3′, and a reverse primer consisting of a 9∶1 mixture of two oligonucleotides, 5′-*B-* AGAGTTTGATCMTGGCTCAG-3′ and 5′-*B*- AGGGTTCGATTCTGGCTCAG-3′, where *B* represents the B linker (5′-CCTATCCCCTGTGTGCCTTGGCAGTCTCAG-3′) followed by the 16S rRNA 8F and 8F-Bif primers, respectively (Table S1) [34].

PCR amplifications (in a volume of 50 µL) were performed using 1× FastStart High Fidelity Reaction Buffer, 1.8 mM MgCl_2_, 1 mM dNTP solution, 5 U FastStart High Fidelity Blend Polymerase (from the High Fidelity PCR System (Roche, Indianapolis, USA)), 0.2 µM reverse primer, 0.2 µM of the barcoded forward primer (unique for each sample) and 1 µL of template DNA. PCR was performed using the following cycle conditions: an initial denaturation at 94°C for 3 min, followed by 25 cycles of denaturation at 94°C for 30 s, annealing at 51°C for 45 s and extension at 72°C for 5 min, and a final elongation step at 72°C for 10 min. Amplicons (20 µL) were purified using AMPure XP purification (Agencourt, Massachusetts, USA) according to the manufacturer’s instructions and eluted in 25 µl 1× low TE (10 mM Tris-HCl, 0.1 mM EDTA, pH 8.0).

Amplicon concentrations were determined by Quant-iT PicoGreen dsDNA reagent kit (Invitrogen, New York, USA*)* using a Victor3 Multilabel Counter (Perkin Elmer, Waltham, USA). Amplicons were mixed in equimolar concentrations to ensure equal representation of each sample. A one-region 454 sequencing run was performed on a GS FLX Titanium PicoTiterPlate with a GS FLX pyrosequencing system (Roche, Branford, USA).

### Data Analysis

The V1–V3 16S rDNA bacterial sequences analyzed in this paper have been deposited in the MG-RAST database (project ID: 4728).

Raw pyrosequencing reads were initially passed through quality filters to reduce the overall error rate using Mothur version 1.23 [35]. Only those sequences with perfect proximal primer fidelity and a threshold quality score of ≥20, a read length between 200 and 540 nucleotides, a maximum of one ambiguous base call and a maximum homopolymer length of 9 were retained for further analysis.

Subsequent data processing was conducted using Quantitative Insights Into Microbial Ecology (QIIME) version 1.5.1 [36]. Barcodes were used to identify sequences belonging to each patient sample. The UCLUST algorithm was used to cluster sequences into operational taxonomic units (OTUs) or phylotypes based on 97% similarity (species level) against the Greengenes reference set [37]. The following nondefault search parameters for UCLUST were applied: maxrejects = 100 and stepwords = 16. Creation of new clusters for sequences that did not cluster to reference sequences within the given similarity threshold was disabled, to further reduce the influence of pyrosequencing errors.

Species richness and diversity within communities (alpha-diversity) was measured by means of the observed OTUs (observed richness), Chao1 index (estimated richness), and the Shannon diversity index. Beta-diversity or diversity shared across patient communities was determined by UniFrac distance and Bray-Curtis dissimilarity (BC). UniFrac distances are based on the fraction of branch length shared between two communities within a phylogenetic tree constructed from the 16S rRNA gene sequences from all communities being compared. A relatively small UniFrac distance implies that two communities are compositionally similar, harboring lineages sharing a common evolutionary history [38]. Both weighted and unweighted UniFrac were used, respectively taking into account the abundance of each bacterial species for creating branch length or not.

### Statistical Analysis

BC and Unweighted UniFrac distances were calculated for each patient (remission versus active sample) and distances were subsequently compared between CD and UC patients using the Mann-Whitney U test. Distances between subsequent samples from the same patient (within subjects) and distances between remission samples of different patients (between subjects) were also compared using the Mann-Whitney U test. The above mentioned statistical analyses were conducted using SPSS version 20.

All subsequent statistical analyses were conducted within QIIME 1.5.1, unless stated otherwise.

Age was dichotomized in two groups with 52 years (median age) as cut-off. Differences in alpha-diversity with respect to IBD type, disease location, age, gender and medication use (thiopurines, TNF-α inhibitors, aminosalicylates, prednisone or methotrexate) were tested using a nonparametric two-sample t-tests (i.e. using Monte Carlo permutations to calculate the p-value) at a rarefaction intensity of >6,190 sequences per sample. To test for differences in alpha-diversity within patients with respect to disease activity, the Wilcoxon Signed-Rank test was applied (conducted within SPSS v. 20).

OTU coverage was estimated using the conditional uncovered probability method [39]. Analysis of similarities (ANOSIM) was used to test for differences in community structure (UniFrac) among the various groups (IBD type, disease location, age, gender and medication use) in remission state. ANOSIM is a permutation-based test of the null hypothesis that within-group distances are not significantly smaller than between-group distances [40].

To test for associations between OTUs (presence) and IBD type, disease location, age, gender and medication use, the G-test statistic was used. To test for differences in the relative abundance of OTUs, the paired-sample T-test was used with respect to disease activity (within group comparisons) and ANOVA was used to test for differences with respect to IBD type, disease location, age, gender and medication use between groups. The false discovery rate (FDR) method was used to correct for multiple testing at a cutoff of 0.25. The relatively high value was used so as not to miss possible associations.

## Results

### Study Population

Out of the 323 patients from the prospective IBD cohort, with an average of three subsequent samples per subject, ten CD and nine UC patients that fulfilled our criteria could be selected. Patient characteristics are presented in Table 1. All patients, except one, had calprotectin levels above 150 µg/g feces at time of active disease. The single patient with a calprotectin level below 150 µg/g, showed a 9-fold increase (from 14 toward 126 µg/g faeces) during the flare as compared to remission.

**Table 1:** Patient characteristics^a.^

	CD (n = 10)	UC (n = 9)
Age (years)	42.5 (22–73)	65.0 (30–79)
Gender	6 ♂, 4 ♀	7 ♂, 2 ♀
Time since diagnosis (years)	7.5 (1–29)	10 (3–28)
Location	3 ileocolonic^c^	2 proctosigmoid
	7 colonic^c^	5 left-sided
		2 pancolitis
Medication use (n) b:		
TNF-α inhibitor	7	1
Thiopurine	4	3
Aminosalicylate	1	4
Methotrexate	1	1
Prednisone	1	1
Calprotectin (µg/g feces):		
- Remission	37.0 (17–82)	15.0 (15–115)
- Activity	365.0 (126–4,900)	231.0 (161–1,120)
Time between samples (remission to activity, in months)	1.5 (1–10)	3.0 (1–6)

a Age (year), calprotectin level (Calp) and time from remission to activity (months) displayed as median with the range in parentheses.

b No change in IBD medication, except doubling of mesalazine in one patient (#5 in subsequent figures and text) and use of lactulose in another patient (#6) during exacerbation.

c Disease extent in colon at time of inclusion was left-sided (n = 5), right sided (n = 2), pancolonic (n = 2) and cecal (n = 2).

### 454 Sequencing

In total, 230,026,772 bases and 551,607 sequences were recovered following pyrosequencing. After trimming, filtering and binning 324,085 sequences (mean length ±SD: 310±72.7 bases), ranging from 6,194 to 11,030 sequences per sample, remained for downstream analysis. A total of 2,839 OTUs were found with an OTU coverage of 97.1% ±1.1% (mean ± s.d.).

**Figure 1:**
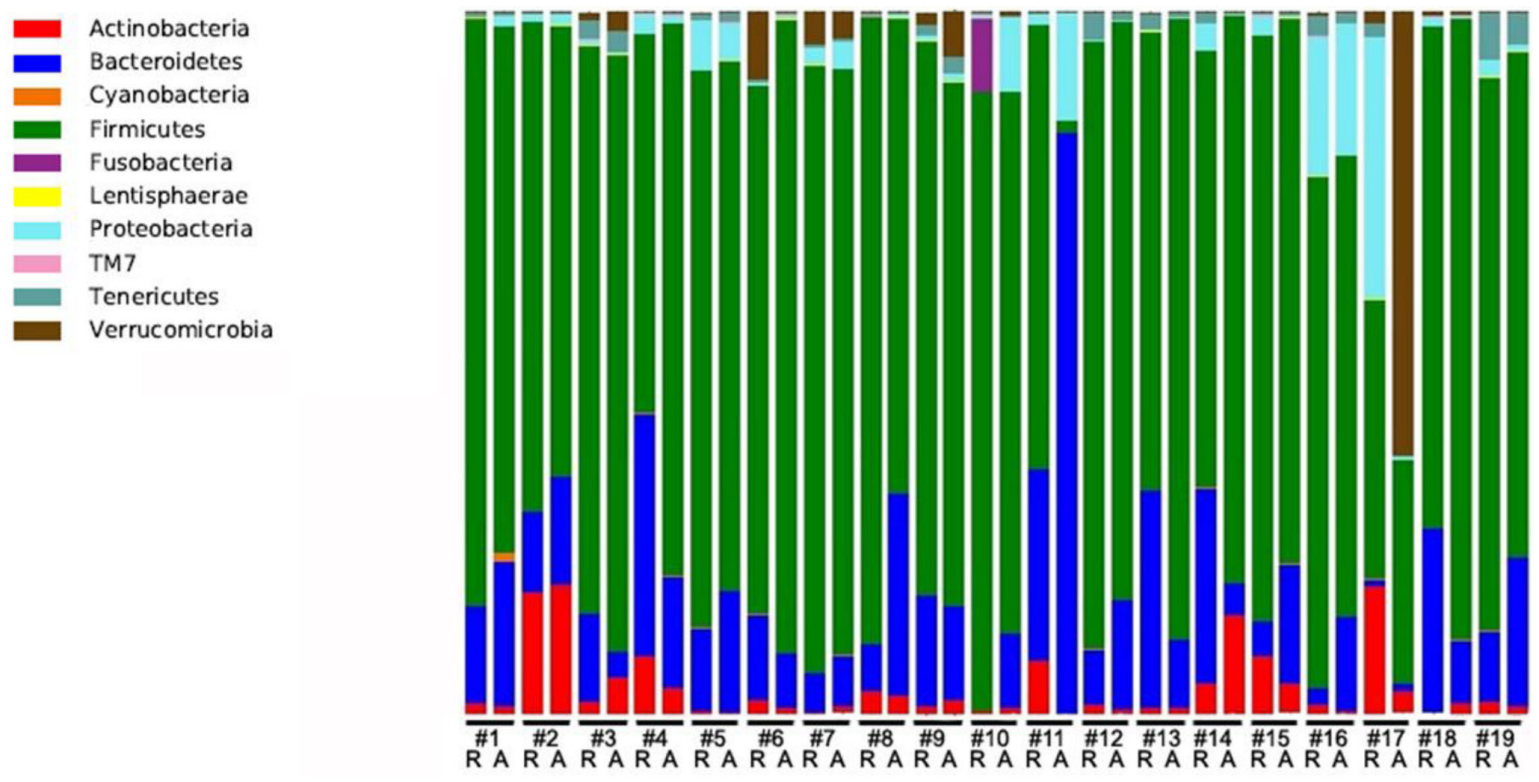
Relative abundance of bacterial phyla in fecal samples of nine UC (#1–9) and ten CD (#10–19) patients during remission and subsequent exacerbation.

Dominant phyla across all samples included Firmicutes (81.3%), Bacteroidetes (15.9%), Proteobacteria (3.8%), Actinobacteria (3.7%), Verrucomicrobia (2.5%) and Tenericutes (1.1%) (Figure 1).

Patient-specific shifts in bacterial composition were observed in all patients. In a subgroup of patients, major shifts were observed for specific bacteria. Two CD patients (labeled #10 and 11 in Figure 1) showed strong increases in the relative abundances of *Bacteroides fragilis* during active disease, i.e. from 0.1% to 10.1% and from 0.1% to 81.9% respectively, whereas another CD patient (#17) showed a large increase in relative abundance of *Akkermansia muciniphila* (1.6% to 62.9%) during activity. Similarly, one UC patient (#8) showed a strong increase in the relative abundance of *Bacteroides dorei* (2.6% to 18.3%) and another UC patient (#3) showed an increase (1.2% to 13.9%) of an undefined *Lactobacillus* species. Despite these shifts in the individual microbial profiles, no significant difference in presence or relative abundance of any particular species or group was detected based on disease activity on a group level neither in the CD nor in the UC patients. Furthermore, no sequences from pathogenic species such as *Campylobacter spp., Helicobacter spp., Salmonella spp.* or *Mycobacterium spp.* were detected.

### Effects on Richness and Diversity

No significant differences in any alpha-diversity metrics were found comparing subgroups based on IBD type, or disease activity (Table 2), nor for disease location, age, gender or use of TNF-α inhibitor, aminosalicylate, prednisone or methotrexate (data not shown). Alpha-diversity seemed to be affected in only one CD patient (#11) during follow up. This patient showed a large relative increase of fecal *B. fragilis* abundance during relapse compared to remission as reported above.

**Table 2:** Alpha-diversity metrics for IBD subtype, disease activity, 6-MP use (Median (range)).

	Reads per sample	Coverage (%)	Observed OTUs	Chao1	Shannon
*CD*	8,053.5	97,4	477.2	684.0	6.6
	(6,503.0–10,033.0)	(94.8–99.3)	(72.1–652.8)	(99.7–1087.6)	(1.4–7.1)
*UC*	8,097.0	97.0	499.3	753.4	6.7
	(6,194–11,030)	(95.8–98.6)	(272.7–675.7)	(433.1–1047.3)	(5.5–7.7)
*Exacerbation*	7,921.0	97.4	493.3	726.3	6.7
	(6,194.0–11,030.0)	(95.8–99.3)	(72.1–664.5)	(99.7–1087.6)	(1.4–7.7)
*Remission*	8,169.0	97.0	493.9	747.7	6.6
	(6,503.0–10,013.0)	(94.8–99.1)	(160.6–675.7)	(308.9–1067.3)	(2.8–7.4)
*Thiopurine use*	8,193.0	97.8	338.9	489.3	5.8
	(6,465.0–10,013.0)	(96.2–99.3)	(72.1–517.4)^a^	(99.7–868.4)^a^	(1.4–7.0)^b^
*No thiopurine* *use*	7,929.0	96.6	569.4	875.9	6.7
	(6,194.0–11,030.0)	(94.8–98.0)	(406.9–675.7)	(605.9–1087.6)	(5.8–7.7)

a significantly lower in thiopurine users, p<0.001.

b significantly lower in thiopurine users, p<0.01.

Thiopurine was used by 7 patients both at remission and during exacerbation (median treatment duration 49.5 (2–182) months), and was found to be associated with a lower number of observed OTUs, Chao1 and Shannon index (Table 2). The median daily dose expressed as 6-mercaptopurine (6-MP) equivalences [41] was 57.9 mg per day.

**Figure 2:**
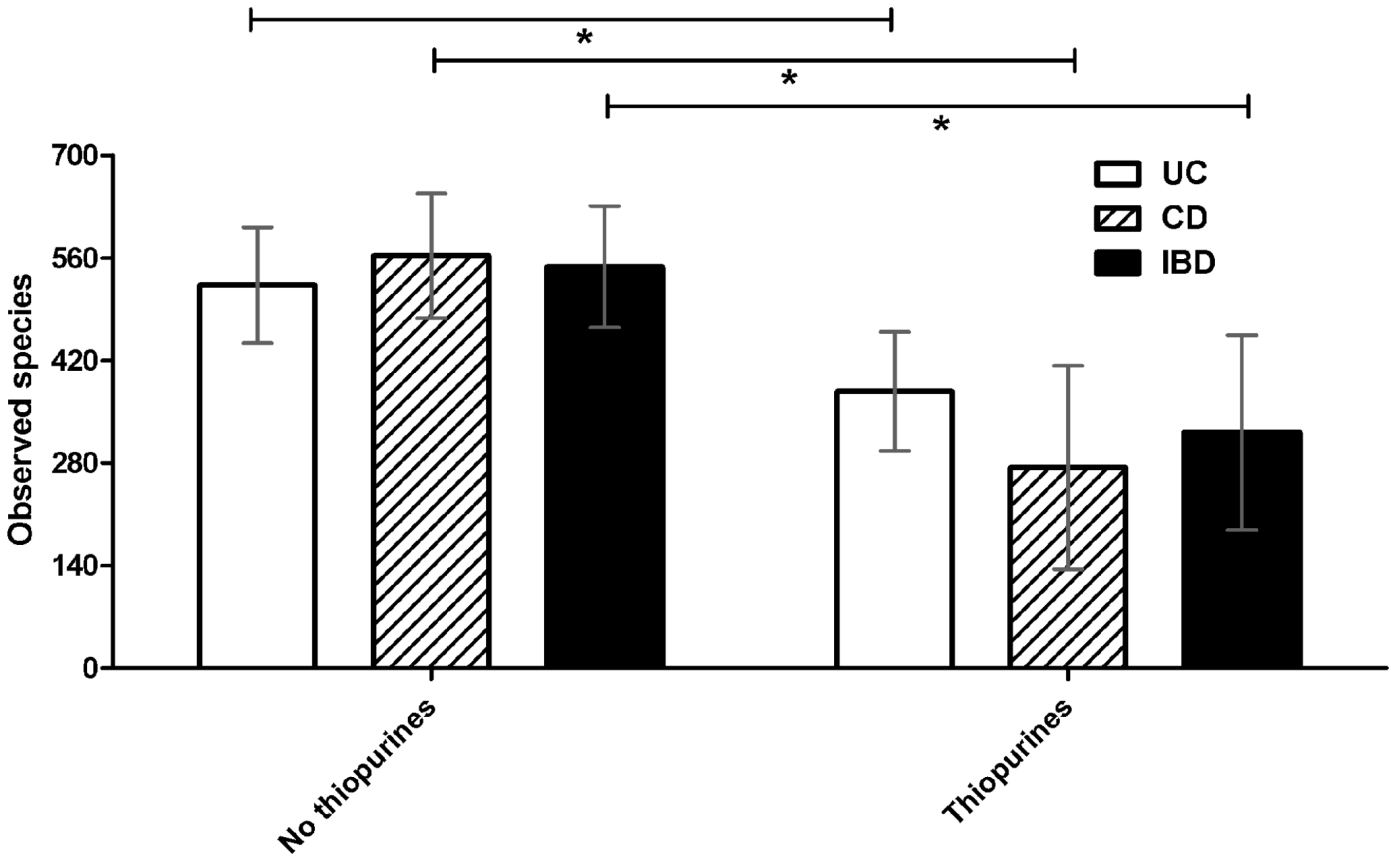
Mean number of observed species (OTUs) according to thiopurine use in Crohn’s disease (p = 2.57*10 ^−**5**^ ), total Inflammatory Bowel Disease (p = 2.37*10 ^−**4**^ ) and Ulcerative Colitis (p = 7.93*10 ^−**4**^ **) patients.** *: Significantly different (p<0.001).

The number of observed OTUs in thiopurine users was significantly decreased among IBD patients in general (p = 2.37 * 10^−4^), but also when studying CD patients (p = 2.57*10^−5^) and UC patients (p = 7.93 * 10^−4^) separately (Figure 2).

### Effects on Community Membership and Composition

**Figure 3:**
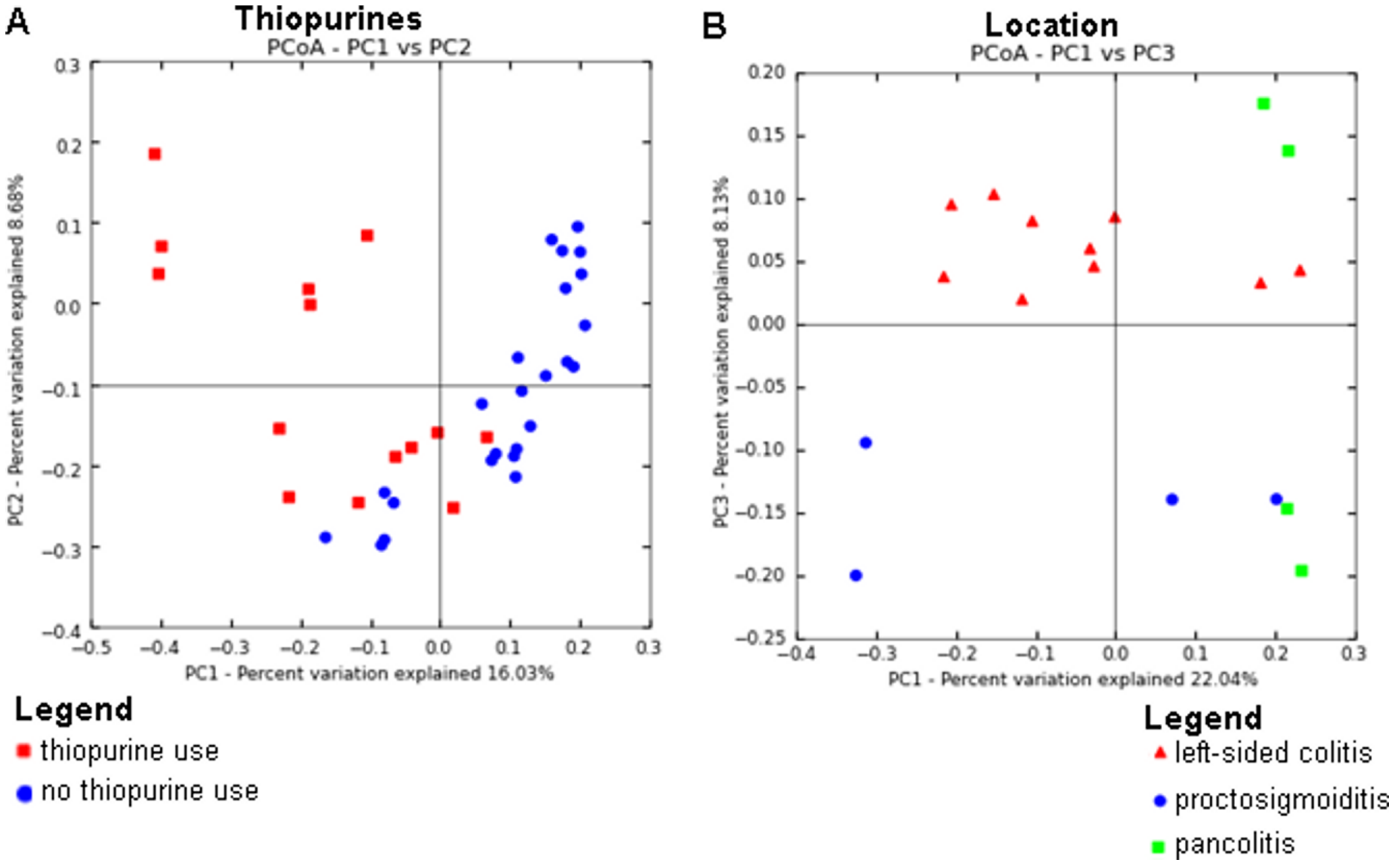
Communities clustered using Principal Coordinates Analysis (PCoA) of the unweighted UniFrac distance matrix. (A) PC1 and PC2 are plotted on *x*- and *y*-axes. Each point corresponds to a community colored according to thiopurine use. All samples are shown. The percentage of variation explained by the plotted principal coordinates is indicated on the axes. (B) PC1 and PC3 are plotted on x- and y-axis. Each point corresponds to a community colored according to disease location. Only samples from UC patients are included.

Unweighted UniFrac-based principal coordinate analysis (PCoA) did not show clustering according to disease activity, IBD type, TNF-α inhibitor, aminosalicylate, prednisone or methotrexate use (Figures S2, S3, S4). Age (R^2^ = 0.16; p = 0.001), thiopurine use (R^2^ = 0.41; p = 0.001), gender (R^2^ = 0.35; p = 0.003), disease location of UC (R^2^ = 0.34; p = 0.008) were related to clustering (Figure 3 and Figure S1), as tested by ANOSIM. For thiopurine use, Fusobacteria and Verrucomicrobia phyla appeared to explain clustering for users, whereas especially Lentisphaerae appeared to determine clustering for non-users (Figure S5).

**Figure 4:**
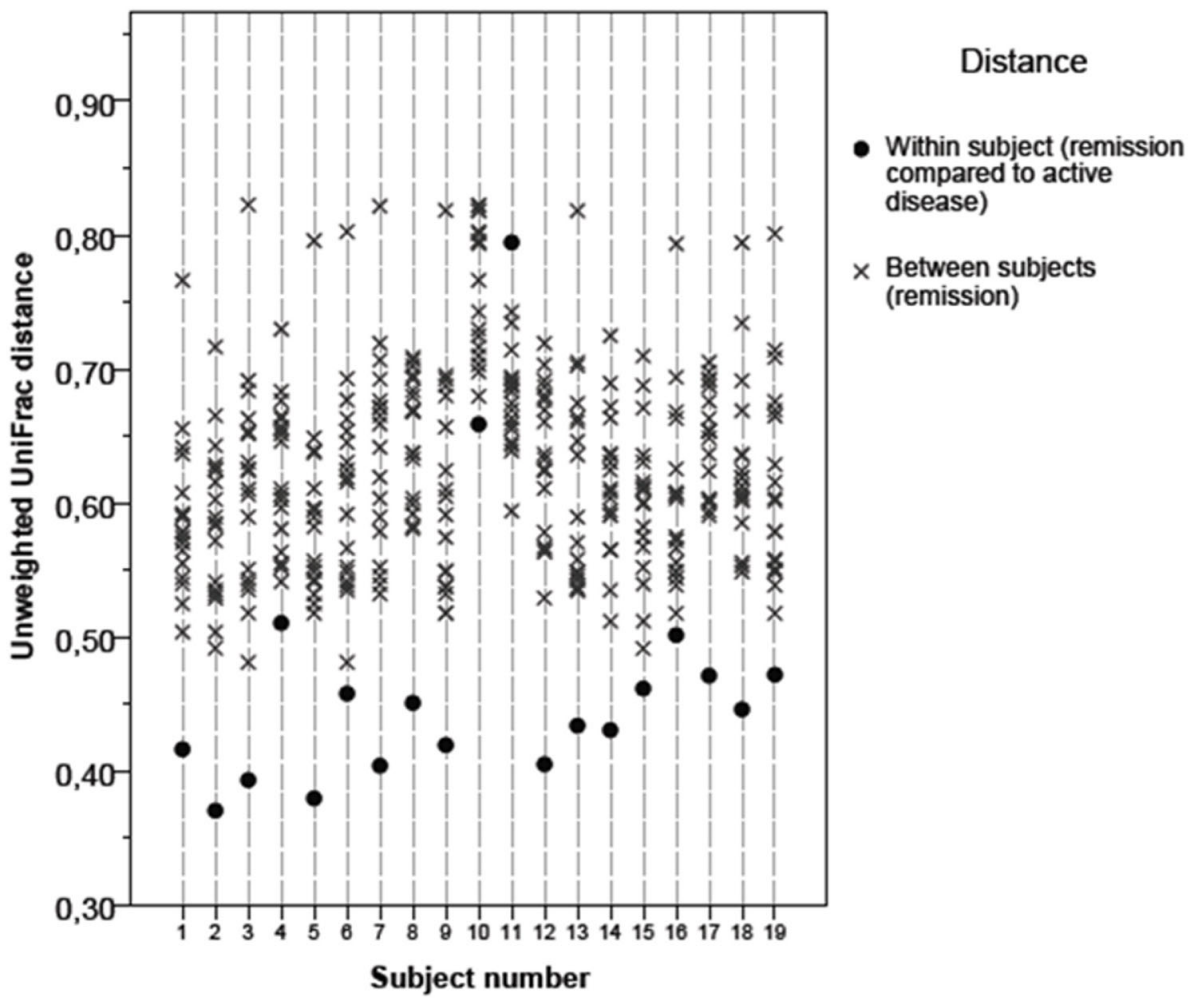
Within and between subjects pair-wise unweighted UniFrac distances for ulcerative colitis (#1–9) and Crohn’s disease (#10–19) patients.

Unweighted UniFrac distances of patients were significantly closer (p<0.001) between their own remission and exacerbation samples than to samples of other patients (Figure 4). Only the remission sample of patient #11 showed more similarity to the other patients’ remission samples than to his own exacerbation sample.

**Figure 5:**
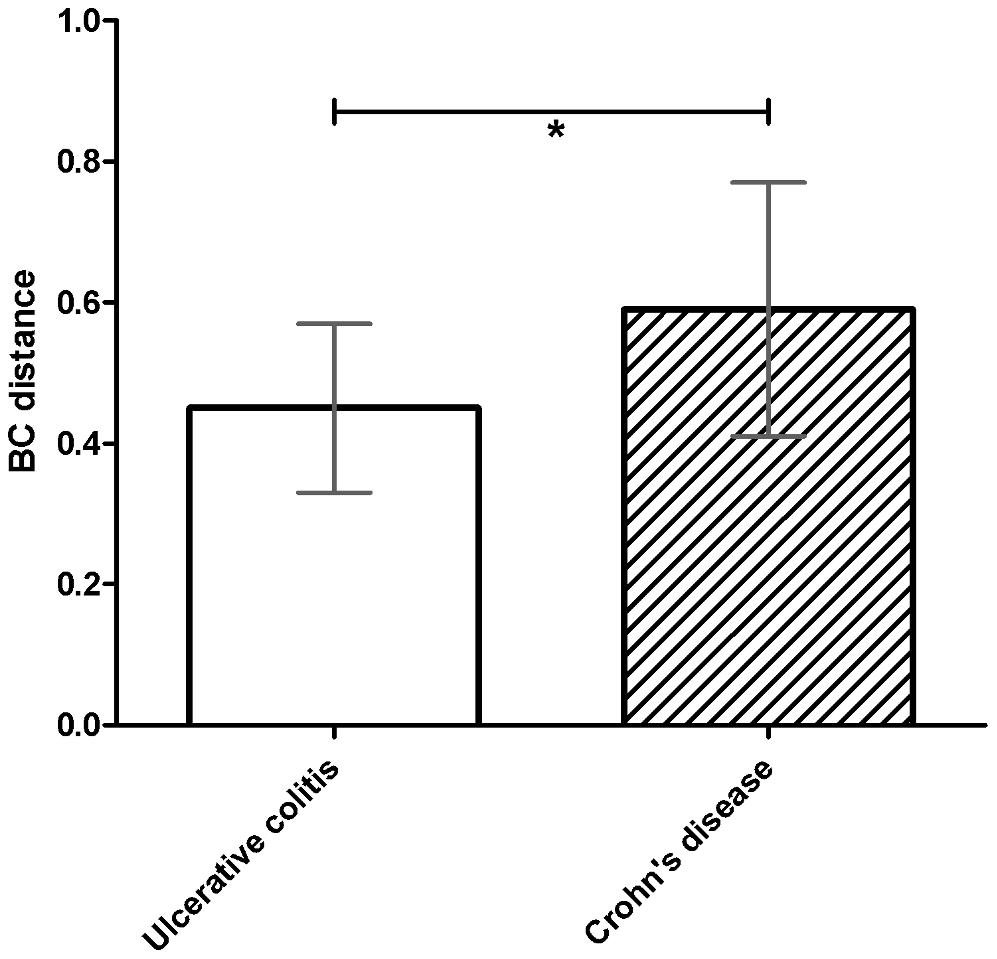
Mean within subjects pair-wise Bray-Curtis distances for ulcerative colitis and Crohn’s disease patients (p = 0.027). *: Significantly different (p<0.05).

Interestingly, the mean distance in community membership between paired Crohn’s disease samples (0.51; s.d. 0.12) was significantly larger as compared to UC samples (0.42; s.d. 0.04; p = 0.027). Using Bray-Curtis dissimilarity distances, the same trend was observed with CD at 0.59 (s.d. 0.18) and UC at 0.42 (s.d. 0.09) (figure 5). This difference between the means was also statistically significant as determined by Mann-Whitney U test with p = 0.027.

Several associations were found between specific OTUs and patient characteristics as determined by G-test and ANOVA, but none of these withstood correction for multiple testing by means of FDR (Tables S2, S3, S4, S5).

## Discussion

To our knowledge, this is the first prospective study using next-generation sequencing to examine the fecal microbiota composition in UC and CD during a quiescent disease phase and a subsequent exacerbation.

Similar to previous studies on the human fecal microbiota, the divisions of Firmicutes and Bacteroidetes predominated [42]–[44]. Although no overarching shifts in microbial communities could be detected in the CD and UC populations, shifts in composition and decreased diversity were observed in individual patients. Moreover, it was demonstrated that the microbiota within CD patients was less stable compared to the microbiota from UC patients. Finally, thiopurines appear to affect the gut microbial composition and diversity.

In the present study, we were not able to identify overall patterns in microbial changes related to the presence of an exacerbation. An exacerbation was defined by endoscopic scores and/or fecal calprotectin levels as indicators for mucosal inflammation, as clinical scores are reported not to correlate very well with endoscopy findings [32], [33]. Mucosal inflammation is increasingly recognized as outcome parameter for active disease and is important for optimization of therapeutic strategies and to prevent complications [45].

Previous studies did show microbial differences comparing active with inactive IBD patients. For example, a combination of leucocytes in the fecal-mucosal transition zone and the concentration of *F. prausnitzii* were found to be strong predictors of active CD and UC [7]. Diminished *F. prausnitzii,* as well as a lower Firmicutes/Bacteroidetes ratio was also associated with active disease in another study on 49 IBD patients [9]. Furthermore, a decrease in the Clostridium family was found in active UC and inactive and active CD compared to inactive UC and healthy controls [20]. A decrease of *C. coccoides*, *C. leptum* subgroup, Atophium cluster and *B. ovatus* was reported in feces of active compared to inactive UC, [8] while increased counts of bifidobacteria, *E. coli* and Clostridia were found in tissue specimens of active compared to inactive UC by others [27].

The above-mentioned studies had a cross-sectional design and are therefore more vulnerable to selection bias and confounding. None of these studies did match patients with quiescent disease to patients with active disease based on characteristics such as disease location, medication, diet and other possible confounders, nor did they adjust for these factors in their statistical analyses. In contrast, two more recent studies that used pyrosequencing did not show clustering based on disease status. A study by Willing et al. used a multivariate statistical approach (partial least-squares discriminant analysis) to distinguish disease phenotypes based on microbial composition in 40 twin pairs [17]. Six patients had active disease based on clinical scores, which could not be distinguished from 34 patients with inactive disease. In the second study, analyzing 231 biopsies and stool samples of IBD patients and healthy subjects, disease activity was not associated with specific shifts in the microbiome composition even after adjusting for other factors such as sample type (i.e. stool or biopsy), IBD type, age, smoking and medication [29].

A potential explanation for the lack of overarching shifts in microbial composition at time of exacerbation in our study may be due to the relatively small study population. Although we included only CD patients with colonic disease and excluded IBD patients with changes in medication over time, some heterogeneity in the study population cannot be excluded. IBD can be considered a set of diseases with overlapping phenotypes rather than a single disease [46], [47]. This may contribute to the patient-specific microbial composition shifts, and lack thereof at group level. The fact that from a large cohort of 323 subjects, only 19 fulfilled our selection criteria, illustrates that it is very difficult to include a rather large homogenous subpopulation.

The main strength of the present study is the prospective design comparing the microbiota in association to disease activity within the same patients followed over time, particularly considering the interindividual differences in microbiota composition. Clear subject-specific changes have been observed. As modifications of medication use over time were not allowed, these changes were most likely associated with the changing disease activity. Major increases (up to 81.9%) in specific bacterial subspecies were observed in five patients, being *Lactobacillus spp*. (in one UC patient), *B. dorei* (in one UC patient), *A. muciniphila* (in one CD patient), and *B. fragilis* (in two CD patients (#10 and #11)). These two latter patients were both non-smoking females (age 35 and 50 years respectively) using a combination of thiopurines and TNF-inhibitors continuously during the period of interest. Apart from developing an exacerbation, no major changes in clinical or environmental factors did occur during the follow-up period in any of these patients that could have contributed to the observed findings. Although larger patient numbers need to be studied before final conclusions can be drawn on the role of the individual commensal in the pathogenesis of IBD, the potential importance of *B. fragilis* is supported by findings from others. *B. fragilis* was found to be significantly more abundant in biopsies from CD patients compared to UC patients and healthy subjects [23]. In another study, the mucosal biofilm mass in IBD patients appeared to be dominated by *B. fragilis* [24]. Some strains of *B. fragilis* are enterotoxigenic by producing a zinc-dependent metalloprotease toxin, and appear to be more prevalent in active IBD patients as compared to inactive patients and control subjects [48]. Animal models have confirmed that enterotoxigenic *B. fragilis* alone is sufficient to induce colitis [49]. Furthermore, a potential role for the eukaryotic-like ubiquitin gene in *B. fragilis* in the pathophysiology of CD has recently been postulated [50].

In the present study, we found a different level of microbial variability contributing to exacerbations in either CD or UC. This may indicate that changes in gut bacterial composition play a more important role in CD than in UC exacerbation development. These findings are in line with findings by others. Willing et al. (2010) found different microbial profiles between CD and controls but not between UC and controls based on 454 sequencing [17]. In addition, Andoh et al. found inactive UC patients to cluster with healthy controls, while inactive and active CD clustered separately together with active UC [20].

Although the numbers were small, we found that thiopurine treatment was associated with the microbial composition and the diversity. Based upon the Principal Coordinate analysis, thiopurine use was found to be the most important factor responsible for clustering and was furthermore associated with a significant decrease in alpha-diversity. It has previously been found that the thiopurines 6-mercaptopurine (6-MP) and azathioprine inhibit growth of *M. avium* subspecies *paratuberculosis in vitro* [51], [52]. We could not demonstrate an antimicrobial effect of 6-MP in vitro as tested on *Bacillus subtilis* by disc diffusion test (test range 1–64 µg, data not shown). This does however not exclude the possibility of an (indirect) effect of 6-MP or one of its metabolites on the intestinal microbiota in vivo.

As such, the present findings indicate that thiopurine use, especially changes in administration or dosage over time, should be considered as a potential confounding factor when studying the microbiota composition in IBD. Replication of our findings on the association between thiopurine use and shifts in microbiota composition as well as more mechanistic insight on the potential underlying mechanisms is warranted to prove a causal relationship.

## Conclusion

Although we did observe patient-specific shifts in microbial composition, we could not demonstrate general changes in microbial composition or diversity in IBD patients at time of exacerbation as compared to a quiescent disease state. Patient-specific shifts in fecal microbial composition seemed to be associated with larger Bray-Curtis dissimilarity between remission and active disease for CD as compared to UC. Furthermore, thiopurine use was found to have significant impact on the microbial composition and diversity, and should be considered when studying the intestinal microbiota in relation to disease course.

Larger prospective studies that enable controlling for potential confounders such as medication, disease subtype and location, are needed to unravel potential general shifts in the microbiota related to exacerbations in IBD.

## Supporting Information

Figure S1 **Communities clustered using Principal Coordinates Analysis (PCoA) of the unweighted UniFrac distance matrix.** (A) PC1 and PC2 are plotted on *x*- and *y*-axes. Each point corresponds to a community colored according to age (red, <52 years; blue ≥52 years). All samples are shown. The percentage of variation explained by the plotted principal coordinates is indicated on the axes. (B) PC1 and PC2 are plotted on x- and y-axis. Each point corresponds to a community colored according to gender (blue, male; red, female).
(TIFF)

Figure S2 **Communities clustered using Principal Coordinates Analysis (PCoA) of the unweighted UniFrac distance matrix.** (A) PC1 and PC2 are plotted on *x*- and *y*-axes. Each point corresponds to a community colored according to aminosalicylate use (red, no aminosalicylate use, blue, aminosalicylate use). All samples are shown. The percentage of variation explained by the plotted principal coordinates is indicated on the axes. (B) PC1 and PC2 are plotted on x- and y-axis. Each point corresponds to a community colored according to IBD type (red, CD; blue UC).
(TIFF)

Figure S3 **Communities clustered using Principal Coordinates Analysis (PCoA) of the unweighted UniFrac distance matrix.** (A) PC1 and PC2 are plotted on *x*- and *y*-axes. Each point corresponds to a community colored according to TNF-α inhibitor use (red, TNF-α inhibitor use; blue, no TNF-α inhibitor use). All samples are shown. The percentage of variation explained by the plotted principal coordinates is indicated on the axes. (B) PC1 and PC2 are plotted on x- and y-axis. Each point corresponds to a community colored according to disease activity (red, exacerbation; blue, remission).
(TIFF)

Figure S4 **Communities clustered using Principal Coordinates Analysis (PCoA) of the unweighted UniFrac distance matrix.** (A) PC1 and PC2 are plotted on *x*- and *y*-axes. Each point corresponds to a community colored according to methotrexate use (red, methotrexate use; blue, no methotrexate use). All samples are shown. The percentage of variation explained by the plotted principal coordinates is indicated on the axes. (B) PC1 and PC2 are plotted on x- and y-axis. Each point corresponds to a community colored according to prednisone use (red, no prednisone use; blue, prednisone use).
(TIFF)

Figure S5 **Communities plotted together with phyla using Principal Coordinates Analysis (PCoA) of the unweighted UniFrac distance matrix.** PC1, PC2 and PC3 are plotted on *x*-, *y*- and *z*-axes. Each point corresponds to a community colored according to thiopurine use (red, thiopurine use; blue, no thiopurine use). Note: Axes were deliberately skewed to most clearly depict relation of clustering to bacterial groups.
(TIFF)

Table S1 **Primers used in this study.**
(DOCX)

Table S2 **Species associations to thiopurine use in resting disease.**
(DOCX)

Table S3 **Species associations to age (<52 years/>52 years) in remission state.**
(DOCX)

Table S4 **Species associations to gender in remission state; F– indicates decreased presence in female subjects.**
(DOCX)

Table S5 **Species associations to IBD type in remission state.**
(DOCX)

## References

[1] Scharl M, Rogler G (2012) Inflammatory bowel disease pathogenesis: what is new? Curr Opin Gastroenterol 28: 301–309. 22573190 10.1097/MOG.0b013e328353e61e

[2] Blumenstein I, Bock H, Weber C, Rambow A, Tacke W, et al (2008) Health care and cost of medication for inflammatory bowel disease in the Rhein-Main region, Germany: a multicenter, prospective, internet-based study. Inflamm Bowel Dis 14: 53–60. 17973301 10.1002/ibd.20257

[3] Buchanan J, Wordsworth S, Ahmad T, Perrin A, Vermeire S, et al (2011) Managing the long term care of inflammatory bowel disease patients: The cost to European health care providers. J Crohns Colitis 5: 301–316. 21683300 10.1016/j.crohns.2011.02.005

[4] van der Valk ME, Mangen MJ, Leenders M, Dijkstra G, van Bodegraven AA, et al (2014) Healthcare costs of inflammatory bowel disease have shifted from hospitalisation and surgery towards anti-TNFalpha therapy: results from the COIN study. Gut. 10.1136/gutjnl-2012-303376 23135759

[5] Talley NJ, Abreu MT, Achkar JP, Bernstein CN, Dubinsky MC, et al. (2011) An evidence-based systematic review on medical therapies for inflammatory bowel disease. Am J Gastroenterol 106 Suppl 1: S2–25; quiz S26. 10.1038/ajg.2011.58 21472012

[6] Frank DN, St Amand AL, Feldman RA, Boedeker EC, Harpaz N, et al (2007) Molecular-phylogenetic characterization of microbial community imbalances in human inflammatory bowel diseases. Proc Natl Acad Sci U S A 104: 13780–13785. 17699621 10.1073/pnas.0706625104 PMC1959459

[7] Swidsinski A, Loening-Baucke V, Vaneechoutte M, Doerffel Y (2008) Active Crohn’s disease and ulcerative colitis can be specifically diagnosed and monitored based on the biostructure of the fecal flora. Inflamm Bowel Dis 14: 147–161. 18050295 10.1002/ibd.20330

[8] Takaishi H, Matsuki T, Nakazawa A, Takada T, Kado S, et al (2008) Imbalance in intestinal microflora constitution could be involved in the pathogenesis of inflammatory bowel disease. Int J Med Microbiol 298: 463–472. 17897884 10.1016/j.ijmm.2007.07.016

[9] Sokol H, Seksik P, Furet JP, Firmesse O, Nion-Larmurier I, et al (2009) Low counts of Faecalibacterium prausnitzii in colitis microbiota. Inflamm Bowel Dis 15: 1183–1189. 19235886 10.1002/ibd.20903

[10] Eckburg PB, Bik EM, Bernstein CN, Purdom E, Dethlefsen L, et al (2005) Diversity of the human intestinal microbial flora. Science 308: 1635–1638. 15831718 10.1126/science.1110591 PMC1395357

[11] Sokol H, Seksik P, Rigottier-Gois L, Lay C, Lepage P, et al (2006) Specificities of the fecal microbiota in inflammatory bowel disease. Inflamm Bowel Dis 12: 106–111. 16432374 10.1097/01.MIB.0000200323.38139.c6

[12] Rehman A, Lepage P, Nolte A, Hellmig S, Schreiber S, et al (2010) Transcriptional activity of the dominant gut mucosal microbiota in chronic inflammatory bowel disease patients. J Med Microbiol 59: 1114–1122. 20522625 10.1099/jmm.0.021170-0

[13] Seksik P, Rigottier-Gois L, Gramet G, Sutren M, Pochart P, et al (2003) Alterations of the dominant faecal bacterial groups in patients with Crohn’s disease of the colon. Gut 52: 237–242. 12524406 10.1136/gut.52.2.237 PMC1774977

[14] Sokol H, Lay C, Seksik P, Tannock GW (2008) Analysis of bacterial bowel communities of IBD patients: what has it revealed? Inflamm Bowel Dis 14: 858–867. 18275077 10.1002/ibd.20392

[15] Martinez-Medina M, Aldeguer X, Gonzalez-Huix F, Acero D, Garcia-Gil LJ (2006) Abnormal microbiota composition in the ileocolonic mucosa of Crohn’s disease patients as revealed by polymerase chain reaction-denaturing gradient gel electrophoresis. Inflamm Bowel Dis 12: 1136–1145. 17119388 10.1097/01.mib.0000235828.09305.0c

[16] Kotlowski R, Bernstein CN, Sepehri S, Krause DO (2007) High prevalence of Escherichia coli belonging to the B2+D phylogenetic group in inflammatory bowel disease. Gut 56: 669–675. 17028128 10.1136/gut.2006.099796 PMC1942160

[17] Willing BP, Dicksved J, Halfvarson J, Andersson AF, Lucio M, et al. (2010) A pyrosequencing study in twins shows that gastrointestinal microbial profiles vary with inflammatory bowel disease phenotypes. Gastroenterology 139: 1844–1854 e1841. 10.1053/j.gastro.2010.08.049 20816835

[18] Nagalingam NA, Lynch SV (2011) Role of the microbiota in inflammatory bowel diseases. Inflamm Bowel Dis. 10.1002/ibd.21866 21936031

[19] Rajilic-Stojanovic M, Shanahan F, Guarner F, de Vos WM (2013) Phylogenetic analysis of dysbiosis in ulcerative colitis during remission. Inflamm Bowel Dis 19: 481–488. 23385241 10.1097/MIB.0b013e31827fec6d

[20] Andoh A, Imaeda H, Aomatsu T, Inatomi O, Bamba S, et al (2011) Comparison of the fecal microbiota profiles between ulcerative colitis and Crohn’s disease using terminal restriction fragment length polymorphism analysis. J Gastroenterol 46: 479–486. 21253779 10.1007/s00535-010-0368-4

[21] Sepehri S, Kotlowski R, Bernstein CN, Krause DO (2007) Microbial diversity of inflamed and noninflamed gut biopsy tissues in inflammatory bowel disease. Inflamm Bowel Dis 13: 675–683. 17262808 10.1002/ibd.20101

[22] Bibiloni R, Mangold M, Madsen KL, Fedorak RN, Tannock GW (2006) The bacteriology of biopsies differs between newly diagnosed, untreated, Crohn’s disease and ulcerative colitis patients. J Med Microbiol 55: 1141–1149. 16849736 10.1099/jmm.0.46498-0

[23] Gophna U, Sommerfeld K, Gophna S, Doolittle WF, Veldhuyzen van Zanten SJ (2006) Differences between tissue-associated intestinal microfloras of patients with Crohn’s disease and ulcerative colitis. J Clin Microbiol 44: 4136–4141. 16988016 10.1128/JCM.01004-06 PMC1698347

[24] Swidsinski A, Weber J, Loening-Baucke V, Hale LP, Lochs H (2005) Spatial organization and composition of the mucosal flora in patients with inflammatory bowel disease. J Clin Microbiol 43: 3380–3389. 16000463 10.1128/JCM.43.7.3380-3389.2005 PMC1169142

[25] Ott SJ, Musfeldt M, Wenderoth DF, Hampe J, Brant O, et al (2004) Reduction in diversity of the colonic mucosa associated bacterial microflora in patients with active inflammatory bowel disease. Gut 53: 685–693. 15082587 10.1136/gut.2003.025403 PMC1774050

[26] Manichanh C, Rigottier-Gois L, Bonnaud E, Gloux K, Pelletier E, et al (2006) Reduced diversity of faecal microbiota in Crohn’s disease revealed by a metagenomic approach. Gut 55: 205–211. 16188921 10.1136/gut.2005.073817 PMC1856500

[27] Mylonaki M, Rayment NB, Rampton DS, Hudspith BN, Brostoff J (2005) Molecular characterization of rectal mucosa-associated bacterial flora in inflammatory bowel disease. Inflamm Bowel Dis 11: 481–487. 15867588 10.1097/01.mib.0000159663.62651.4f

[28] Ohkusa T, Sato N, Ogihara T, Morita K, Ogawa M, et al (2002) Fusobacterium varium localized in the colonic mucosa of patients with ulcerative colitis stimulates species-specific antibody. J Gastroenterol Hepatol 17: 849–853. 12164960 10.1046/j.1440-1746.2002.02834.x

[29] Morgan XC, Tickle TL, Sokol H, Gevers D, Devaney KL, et al (2012) Dysfunction of the intestinal microbiome in inflammatory bowel disease and treatment. Genome Biol 13: R79. 23013615 10.1186/gb-2012-13-9-r79 PMC3506950

[30] Masclee GM, Penders J, Pierik M, Wolffs P, Jonkers D (2013) Enteropathogenic Viruses: Triggers for Exacerbation in IBD? A Prospective Cohort Study Using Real-time Quantitative Polymerase Chain Reaction. Inflamm Bowel Dis 19: 124–131. 22508685 10.1002/ibd.22976

[31] Lennard-Jones JE (1989) Classification of inflammatory bowel disease. Scand J Gastroenterol Suppl 170: 2–6; discussion 16–19. 10.3109/00365528909091339 2617184

[32] af Bjorkesten CG, Nieminen U, Turunen U, Arkkila P, Sipponen T, et al (2012) Surrogate markers and clinical indices, alone or combined, as indicators for endoscopic remission in anti-TNF-treated luminal Crohn’s disease. Scand J Gastroenterol 47: 528–537. 22356594 10.3109/00365521.2012.660542

[33] Regueiro M, Kip KE, Schraut W, Baidoo L, Sepulveda AR, et al (2011) Crohn’s disease activity index does not correlate with endoscopic recurrence one year after ileocolonic resection. Inflamm Bowel Dis 17: 118–126. 20848538 10.1002/ibd.21355

[34] Dethlefsen L, Huse S, Sogin ML, Relman DA (2008) The pervasive effects of an antibiotic on the human gut microbiota, as revealed by deep 16S rRNA sequencing. PLoS Biol 6: e280. 19018661 10.1371/journal.pbio.0060280 PMC2586385

[35] Schloss PD, Westcott SL, Ryabin T, Hall JR, Hartmann M, et al (2009) Introducing mothur: open-source, platform-independent, community-supported software for describing and comparing microbial communities. Appl Environ Microbiol 75: 7537–7541. 19801464 10.1128/AEM.01541-09 PMC2786419

[36] Caporaso JG, Kuczynski J, Stombaugh J, Bittinger K, Bushman FD, et al (2010) QIIME allows analysis of high-throughput community sequencing data. Nat Methods 7: 335–336. 20383131 10.1038/nmeth.f.303 PMC3156573

[37] McDonald D, Price MN, Goodrich J, Nawrocki EP, DeSantis TZ, et al (2011) An improved Greengenes taxonomy with explicit ranks for ecological and evolutionary analyses of bacteria and archaea. ISME J 6: 610–618. 22134646 10.1038/ismej.2011.139 PMC3280142

[38] Lozupone C, Knight R (2005) UniFrac: a new phylogenetic method for comparing microbial communities. Appl Environ Microbiol 71: 8228–8235. 16332807 10.1128/AEM.71.12.8228-8235.2005 PMC1317376

[39] Lladser ME, Gouet R, Reeder J (2011) Extrapolation of urn models via poissonization: accurate measurements of the microbial unknown. PLoS One 6: e21105. 21738613 10.1371/journal.pone.0021105 PMC3125174

[40] Clarke K (1993) Non-parametric multivariate analyses of changes in community structure. Austral J Ecol 18: 117–143.

[41] Sandborn WJ (2001) Rational dosing of azathioprine and 6-mercaptopurine. Gut 48: 591–592. 11302950 10.1136/gut.48.5.591 PMC1728293

[42] Nam YD, Jung MJ, Roh SW, Kim MS, Bae JW (2011) Comparative analysis of Korean human gut microbiota by barcoded pyrosequencing. PLoS One 6: e22109. 21829445 10.1371/journal.pone.0022109 PMC3146482

[43] Claesson MJ, O’Sullivan O, Wang Q, Nikkila J, Marchesi JR, et al (2009) Comparative analysis of pyrosequencing and a phylogenetic microarray for exploring microbial community structures in the human distal intestine. PLoS One 4: e6669. 19693277 10.1371/journal.pone.0006669 PMC2725325

[44] van den Bogert B, de Vos WM, Zoetendal EG, Kleerebezem M (2011) Microarray analysis and barcoded pyrosequencing provide consistent microbial profiles depending on the source of human intestinal samples. Appl Environ Microbiol 77: 2071–2080. 21257804 10.1128/AEM.02477-10 PMC3067328

[45] Vaughn BP, Shah S, Cheifetz AS (2014) The Role of Mucosal Healing in the Treatment of Patients With Inflammatory Bowel Disease. Curr Treat Options Gastroenterol. 10.1007/s11938-013-0008-1 PMC3938312 24395615

[46] Kaser A, Zeissig S, Blumberg RS (2010) Genes and environment: how will our concepts on the pathophysiology of IBD develop in the future? Dig Dis 28: 395–405. 20926863 10.1159/000320393 PMC2980818

[47] Young VB, Kahn SA, Schmidt TM, Chang EB (2011) Studying the Enteric Microbiome in Inflammatory Bowel Diseases: Getting through the Growing Pains and Moving Forward. Front Microbiol 2: 144. 21772835 10.3389/fmicb.2011.00144 PMC3131521

[48] Prindiville TP, Sheikh RA, Cohen SH, Tang YJ, Cantrell MC, et al (2000) Bacteroides fragilis enterotoxin gene sequences in patients with inflammatory bowel disease. Emerg Infect Dis 6: 171–174. 10756151 10.3201/eid0602.000210 PMC2640860

[49] Rabizadeh S, Rhee KJ, Wu S, Huso D, Gan CM, et al (2007) Enterotoxigenic bacteroides fragilis: a potential instigator of colitis. Inflamm Bowel Dis 13: 1475–1483. 17886290 10.1002/ibd.20265 PMC3056612

[50] Patrick S, Blakely GW (2012) Crossing the eukaryote-prokaryote divide: A ubiquitin homolog in the human commensal bacterium Bacteroides fragilis. Mob Genet Elements 2: 149–151. 23061022 10.4161/mge.21191 PMC3463472

[51] Greenstein RJ, Su L, Haroutunian V, Shahidi A, Brown ST (2007) On the action of methotrexate and 6-mercaptopurine on M. avium subspecies paratuberculosis. PLoS One 2: e161. 17252054 10.1371/journal.pone.0000161 PMC1779805

[52] Shin SJ, Collins MT (2008) Thiopurine drugs azathioprine and 6-mercaptopurine inhibit Mycobacterium paratuberculosis growth in vitro. Antimicrob Agents Chemother 52: 418–426. 18070971 10.1128/AAC.00678-07 PMC2224720

