# Supplementary figures and images for: Fecal Microbial Composition of Ulcerative Colitis and Crohn’s Disease Patients in Remission and Subsequent Exacerbation
Source: PLoS One. 2014 Mar 7;9(3):e90981. doi: 10.1371/journal.pone.0090981 (PMC3946581; doi:10.1371/journal.pone.0090981)

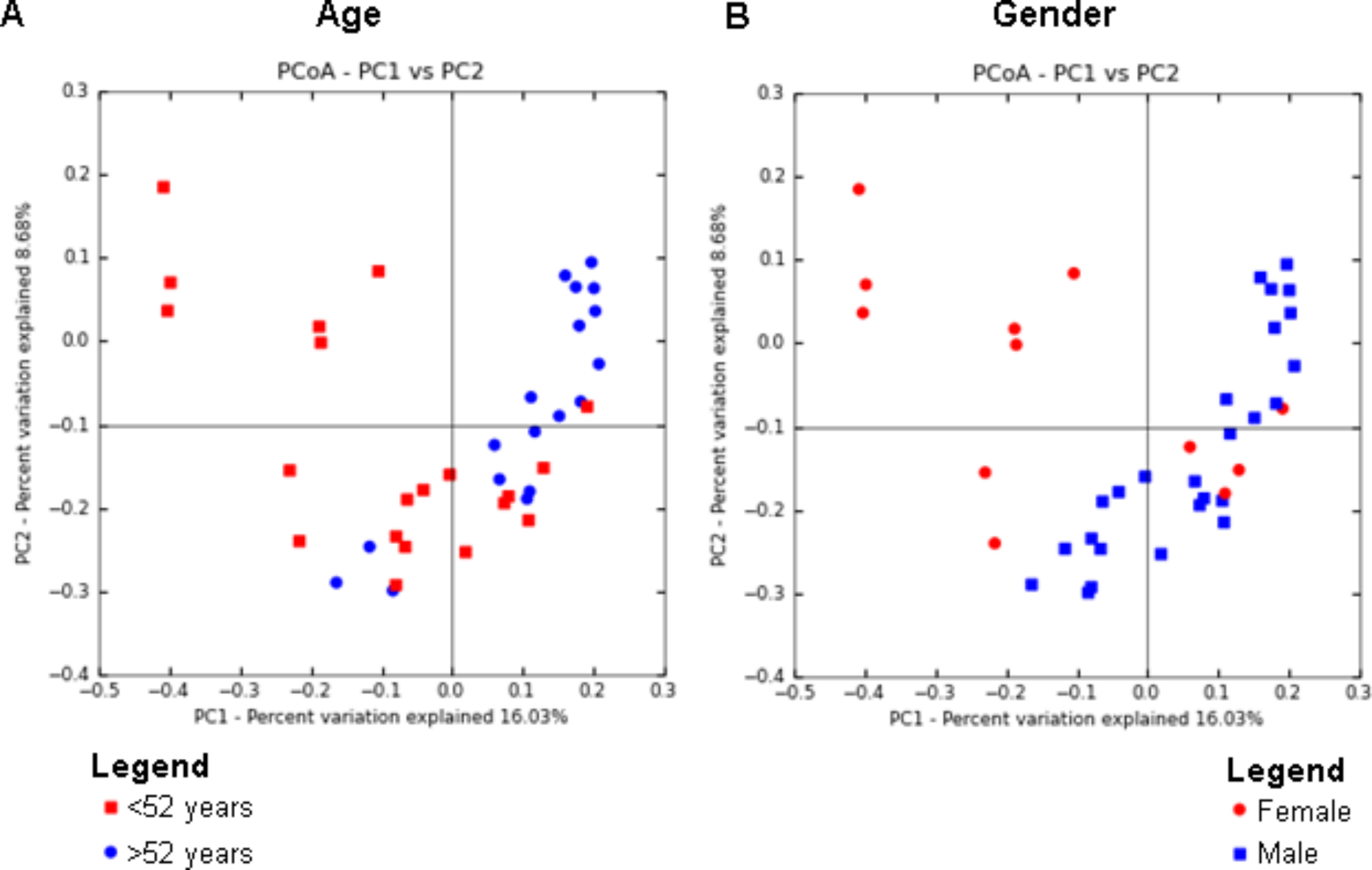

Supplement: Figure S1 — Communities clustered using Principal Coordinates Analysis (PCoA) of the unweighted UniFrac distance matrix. (A) PC1 and PC2 are plotted on x- and y-axes. Each point corresponds to a community colored according to age (red, <52 years; blue ≥52 years). All samples are shown. The percentage of variation explained by the plotted principal coordinates is indicated on the axes. (B) PC1 and PC2 are plotted on x- and y-axis. Each point corresponds to a community colored according to gender (blue, male; red, female). (TIFF) [file pone.0090981.s001.tif]

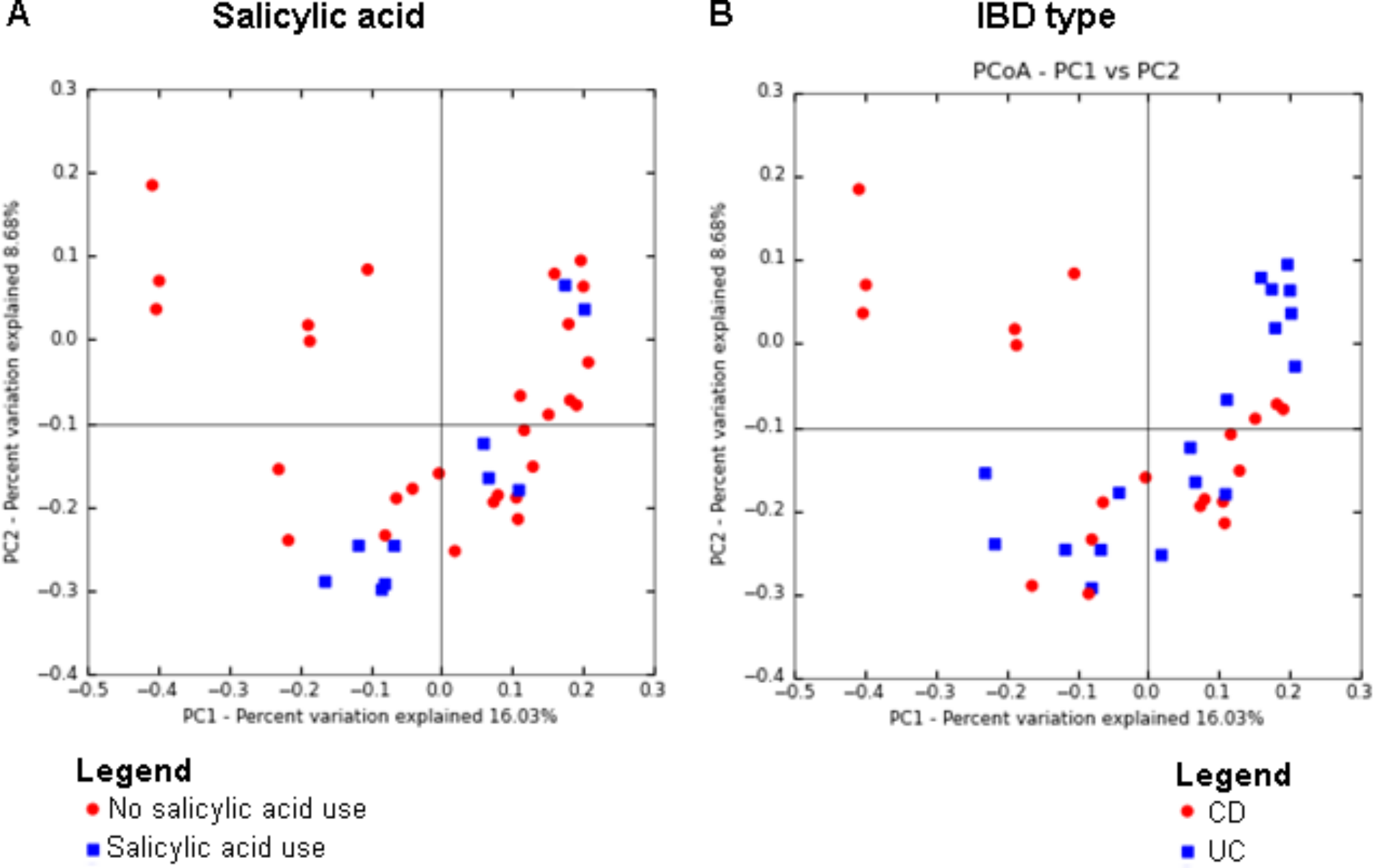

Supplement: Figure S2 — Communities clustered using Principal Coordinates Analysis (PCoA) of the unweighted UniFrac distance matrix. (A) PC1 and PC2 are plotted on x- and y-axes. Each point corresponds to a community colored according to aminosalicylate use (red, no aminosalicylate use, blue, aminosalicylate use). All samples are shown. The percentage of variation explained by the plotted principal coordinates is indicated on the axes. (B) PC1 and PC2 are plotted on x- and y-axis. Each point corresponds to a community colored according to IBD type (red, CD; blue UC). (TIFF) [file pone.0090981.s002.tif]

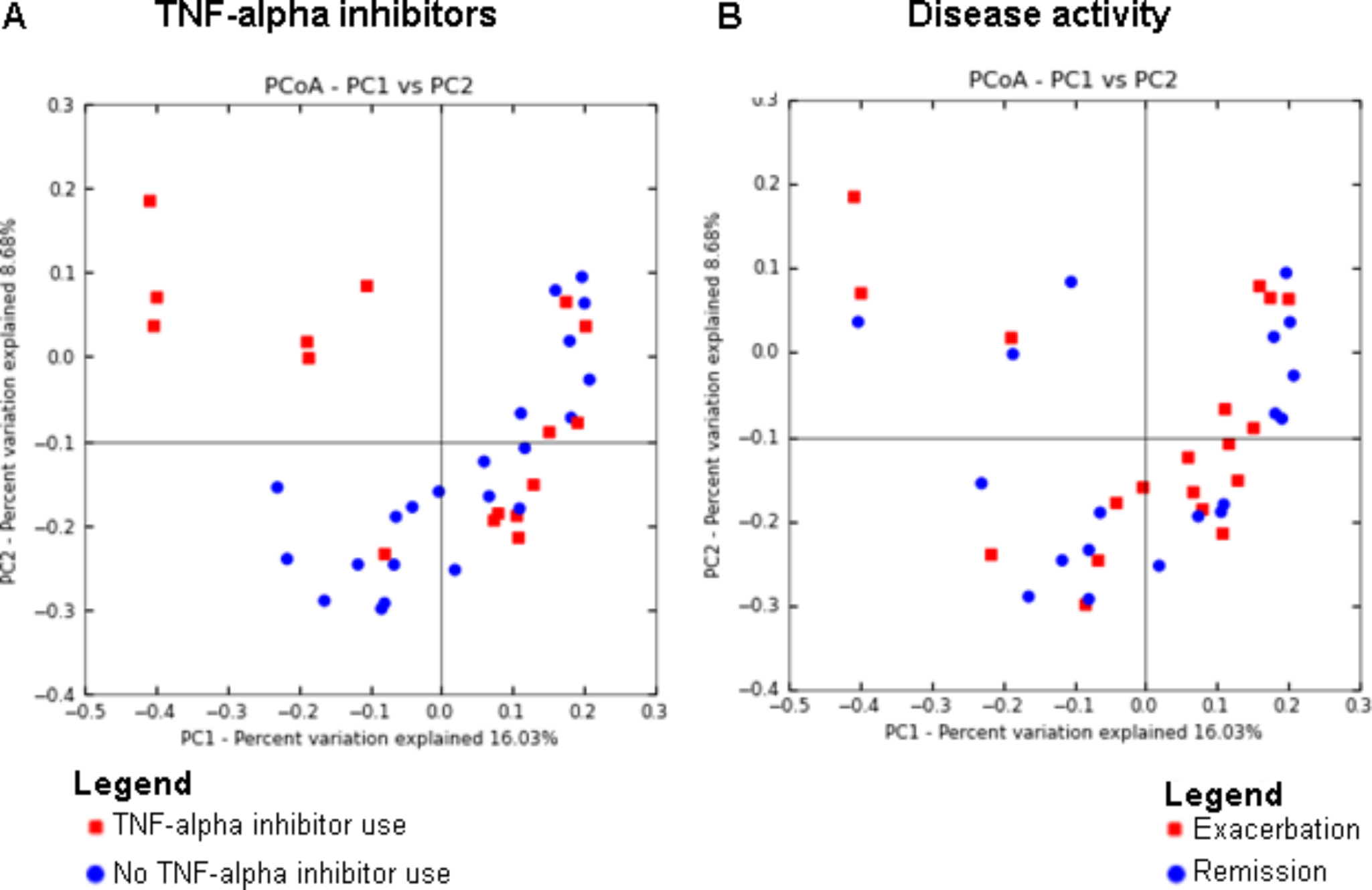

Supplement: Figure S3 — Communities clustered using Principal Coordinates Analysis (PCoA) of the unweighted UniFrac distance matrix. (A) PC1 and PC2 are plotted on x- and y-axes. Each point corresponds to a community colored according to TNF-α inhibitor use (red, TNF-α inhibitor use; blue, no TNF-α inhibitor use). All samples are shown. The percentage of variation explained by the plotted principal coordinates is indicated on the axes. (B) PC1 and PC2 are plotted on x- and y-axis. Each point corresponds to a community colored according to disease activity (red, exacerbation; blue, remission). (TIFF) [file pone.0090981.s003.tif]

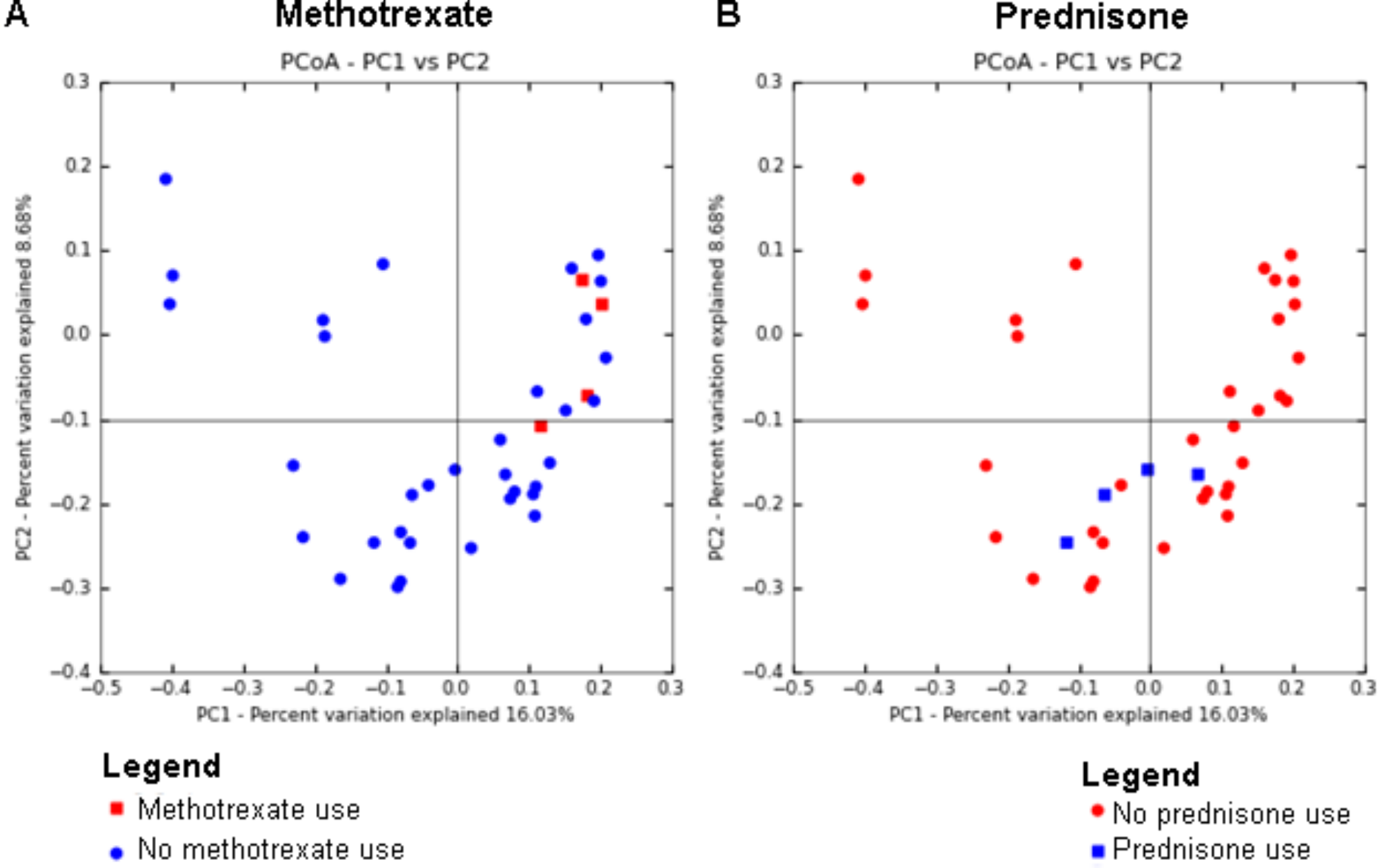

Supplement: Figure S4 — Communities clustered using Principal Coordinates Analysis (PCoA) of the unweighted UniFrac distance matrix. (A) PC1 and PC2 are plotted on x- and y-axes. Each point corresponds to a community colored according to methotrexate use (red, methotrexate use; blue, no methotrexate use). All samples are shown. The percentage of variation explained by the plotted principal coordinates is indicated on the axes. (B) PC1 and PC2 are plotted on x- and y-axis. Each point corresponds to a community colored according to prednisone use (red, no prednisone use; blue, prednisone use). (TIFF) [file pone.0090981.s004.tif]

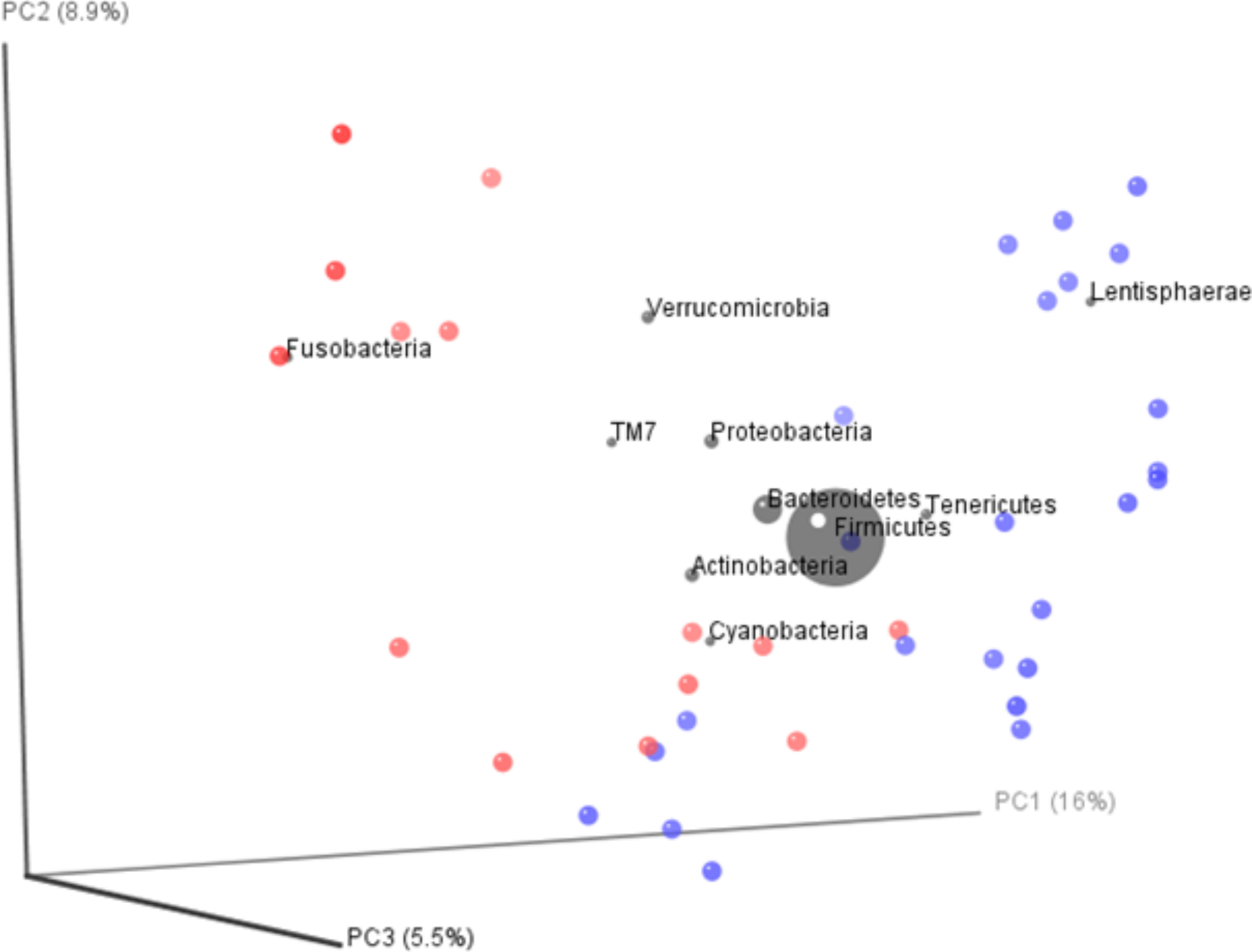

Supplement: Figure S5 — Communities plotted together with phyla using Principal Coordinates Analysis (PCoA) of the unweighted UniFrac distance matrix. PC1, PC2 and PC3 are plotted on x-, y- and z-axes. Each point corresponds to a community colored according to thiopurine use (red, thiopurine use; blue, no thiopurine use). Note: Axes were deliberately skewed to most clearly depict relation of clustering to bacterial groups. (TIFF) [file pone.0090981.s005.tif]
