# Supplementary material for: Fecal Microbial Composition of Ulcerative Colitis and Crohn’s Disease Patients in Remission and Subsequent Exacerbation
Source: PLoS One. 2014 Mar 7;9(3):e90981. doi: 10.1371/journal.pone.0090981 (PMC3946581; doi:10.1371/journal.pone.0090981)
Supplement: Table S1 — Primers used in this study. (DOCX) [file pone.0090981.s006.docx]

Table S1: Primers used in this study

| Forward primers (5’-3’): |  |
| --- | --- |
|  | **Sequences** |
| **454 A linker:**barcode:515R primer | **CCATCTCATCCCTGCGTGTCTCCGACTCAG***NNNNNN*ATTACCGCGGCTGCTGG |
| Barcode 1 | CACGC |
| Barcode 2 | CGCAAC |
| Barcode 3 | TGAAGC |
| Barcode 4 | ACTTGC |
| Barcode 5 | TCACAC |
| Barcode 6 | CGTGAC |
| Barcode 7 | ACGCGC |
| Barcode 8 | CCTCTC |
| Barcode 9 | ACTCAC |
| Barcode 10 | AGACAC |
| Barcode 11 | CGACTC |
| Barcode 12 | AGCTTC |
| Barcode 13 | AAGCCGC |
| Barcode 14 | CAAGAAC |
| Barcode 15 | AGTTGGC |
| Barcode 16 | TATCAAC |
| Barcode 17 | AGGCGGC |
| Barcode 18 | CGGTATC |
| Barcode 19 | TGACGAC |
| Barcode 20 | ACAAGGC |
| Barcode 21 | AGACCTC |
| Barcode 22 | ATACCAC |
| Barcode 23 | TCGCGGC |
| Barcode 24 | ATCTTAC |
| Barcode 25 | AACCAGC |
| Barcode 26 | TTCGAGC |
| Barcode 27 | AAGGTGC |
| Barcode 28 | TCTTGGC |
| Barcode 29 | TAATCTC |
| Barcode 30 | TCACCTC |
| Barcode 31 | TCTCGAC |
| Barcode 32 | CCAGGAC |
| Barcode 33 | ACTCCTC |
| Barcode 34 | TTCCTGC |
| Barcode 35 | TCCGCTC |
| Barcode 36 | TTCATAC |
| Barcode 37 | AGTCGAC |
| Barcode 38 | TATTGAC |
| Barcode 39 | TGCGTTC |
| Barcode 40 | ACGGCTC |
| **Reverse primers (5’- 3’):** | **Sequences** |
| **454 B linker**:8f primer | **CCTATCCCCTGTGTGCCTTGGCAGTCTCAG**AGAGTTTGATCMTGGCTCAG |
| **454 B linker**:8f-bif primer | **CCTATCCCCTGTGTGCCTTGGCAGTCTCAG**AGGGTTCGATTCTGGCTCAG |
