# Supplementary material for: Fecal Microbial Composition of Ulcerative Colitis and Crohn’s Disease Patients in Remission and Subsequent Exacerbation
Source: PLoS One. 2014 Mar 7;9(3):e90981. doi: 10.1371/journal.pone.0090981 (PMC3946581; doi:10.1371/journal.pone.0090981)
Supplement: Table S2 — Species associations to thiopurine use in resting disease. (DOCX) [file pone.0090981.s007.docx]

Table S2: Species associations to thiopurine use in resting disease

| **Species** | **Effect of thiopurines** | **P-value. uncorrected** | **Q-value. FDR** |
| --- | --- | --- | --- |
| ***Dialister invisus*** | + | 6.42 * 10^-4^ | 0.48 |
| **Lachnospiraceae undefined genus** | – | 6.56 * 10^-3^ | 2.44 |
| **Lachnospiraceae undefined genus** | – | 6.56 * 10^-2^ | 1.62 |
| **Ruminococcaceae undefined genus** | – | 1.74 * 10^-2^ | 3.23 |
| **Clostridiales undefined family** | – | 1.74 * 10^-2^ | 2.58 |
| ***Faecalibacterium* undefined species** | – | 1.74 * 10^-2^ | 2.15 |
| ***Oscillospira* undefined species** | – | 1.74 * 10^-2^ | 1.84 |
| **Ruminococcaceae undefined genus** | – | 1.74 * 10^-2^ | 1.61 |
| ***Faecalibacterium* undefined species** | – | 2.25 * 10^-2^ | 1.86 |
| ***Faecalibacterium prausnitzii*** | – | 2.25 * 10^-2^ | 1.67 |
| ***Faecalibacterium prausnitzii*** | – | 2.25 * 10^-2^ | 1.52 |
| ***Lachnobacterium* undefined species** | – | 2.25 * 10^-2^ | 1.39 |
| ***Faecalibacterium* undefined species** | – | 2.25 * 10^-2^ | 1.28 |
| ***Faecalibacterium* undefined species** | – | 2.25 * 10^-2^ | 1.19 |
| ***Faecalibacterium* undefined species** | – | 2.25 * 10^-2^ | 1.11 |
| **Lachnospiraceae undefined genus** | – | 2.25 * 10^-2^ | 1.05 |
| **Lachnospiraceae undefined genus** | – | 3.04 * 10^-2^ | 1.33 |
| **Ruminococcaceae undefined genus** | – | 3.04 * 10^-2^ | 1.25 |
| ***Bacteroides ovatus*** | – | 3.04 * 10^-2^ | 1.19 |
| **Lachnospiraceae undefined genus** | – | 3.04 * 10^-2^ | 1.13 |
