# Supplementary material for: Fecal Microbial Composition of Ulcerative Colitis and Crohn’s Disease Patients in Remission and Subsequent Exacerbation
Source: PLoS One. 2014 Mar 7;9(3):e90981. doi: 10.1371/journal.pone.0090981 (PMC3946581; doi:10.1371/journal.pone.0090981)
Supplement: Table S3 — Species associations to age (<52 years/>52 years) in remission state. (DOCX) [file pone.0090981.s008.docx]

Table S3: Species associations to age (<52 years / >52 years) in remission state

| **Species** | **Effect of age** | **P-value. uncorrected** | **Q-value. FDR** |
| --- | --- | --- | --- |
| **Lachnospiraceae undefined genus** | + | 1.66 * 10^-3^ | 1.23 |
| **Ruminococcaceae undefined genus** | + | 1.97 * 10^-3^ | 0.73 |
| ***Oscillospira* undefined species** | + | 1.97 * 10^-3^ | 0.49 |
| **Lachnospiraceae undefined genus** | + | 6.56 * 10^-3^ | 1.21 |
| ***Coprococcus* undefined species** | + | 6.56 * 10^-3^ | 0.97 |
| ***Blautia* undefined species** | + | 2.03 * 10^-3^ | 2.51 |
| ***Coprococcus* undefined species** | + | 2.03 * 10^-3^ | 2.15 |
| **Ruminoccocaceae undefined genus** | + | 2.03 * 10^-3^ | 1.88 |
| **Lachnospiraceae undefined genus** | + | 2.03 * 10^-3^ | 1.67 |
| ***Coprococcus* undefined species** | + | 2.24 * 10^-3^ | 1.67 |
| **Lachnospiraceae undefined genus** | + | 2.43 * 10^-3^ | 1.64 |
| **Clostridiales undefined family** | + | 2.43 * 10^-3^ | 1.50 |
| ***Ruminococcus* undefined species** | + | 2.43 * 10^-3^ | 1.38 |
| **Prevotellacea undefined genus** | + | 2.76 * 10^-3^ | 1.46 |
| ***Oscillospira* undefined species** | + | 2.76 * 10^-3^ | 1.36 |
| ***Roseburia* undefined species** | + | 2.76 * 10^-3^ | 1.27 |
| **Ruminococcaceae undefined genus** | + | 2.76 * 10^-3^ | 1.20 |
| **Clostridiales undefined family** | + | 2.76 * 10^-3^ | 1.14 |
| ***Roseburia faecis*** | + | 2.76 * 10^-3^ | 1.08 |
| ***Lachnospira* undefined species** | + | 2.76 * 10^-3^ | 1.02 |
