# Supplementary material for: Fecal Microbial Composition of Ulcerative Colitis and Crohn’s Disease Patients in Remission and Subsequent Exacerbation
Source: PLoS One. 2014 Mar 7;9(3):e90981. doi: 10.1371/journal.pone.0090981 (PMC3946581; doi:10.1371/journal.pone.0090981)
Supplement: Table S4 — Species associations to gender in remission state; F– indicates decreased presence in female subjects. (DOCX) [file pone.0090981.s009.docx]

Table S4: Species associations to gender in remission state; F– indicates decreased presence in female subjects

| **Species** | **Effect of gender** | **P-value. uncorrected** | **Q-value. FDR** |
| --- | --- | --- | --- |
| ***Faecalibacterium* undefined species** | F – | 9.67 * 10^-3^ | 0.80 |
| ***Lachnobacterium* undefined species** | F – | 9.67 * 10^-3^ | 0.90 |
| ***Faecalibacterium* undefined species** | F – | 9.67 * 10^-3^ | 1.02 |
| **Lachnospiraceae undefined genus** | F – | 9.67 * 10^-3^ | 1.20 |
| ***Faecalibacterium* undefined species** | F – | 9.67 * 10^-3^ | 1.43 |
| ***Faecalibacterium* undefined species** | F – | 9.67 * 10^-3^ | 1.79 |
| ***Faecalibacterium* undefined species** | F – | 9.67 * 10^-3^ | 2.39 |
| ***Faecalibacterium* undefined species** | F – | 9.67 * 10^-3^ | 3.59 |
| ***Faecalibacterium* undefined species** | F – | 9.67 * 10^-3^ | 7.17 |
| ***Faecalibacterium* undefined species** | F – | 1.30 * 10^-2^ | 0.88 |
| ***Oscillospira* undefined species** | F – | 1.30 * 10^-2^ | 0.97 |
| ***Faecalibacterium* undefined species** | F – | 4.24 * 10^-2^ | 2.62 |
| ***Faecalibacterium* undefined species** | F – | 4.43 * 10^-2^ | 1.73 |
| **Lachnospiraceae undefined genus** | F – | 4.43 * 10^-2^ | 1.83 |
| **Lachnospiraceae undefined genus** | F – | 4.43 * 10^-2^ | 1.93 |
| **Lachnospiraceae undefined genus** | F – | 4.43 * 10^-2^ | 2.05 |
| ***Faecalibacterium* undefined species** | F – | 4.43 * 10^-2^ | 2.19 |
| ***Coprococcus* undefined species** | F – | 4.43 * 10^-2^ | 2.35 |
| ***Faecalibacterium* undefined species** | F – | 4.43 * 10^-2^ | 2.52 |
| ***Blautia* undefined species** | F – | 4.79 * 10^-2^ | 1.69 |
| ***Faecalibacterium* undefined species** | F – | 4.79 * 10^-2^ | 1.78 |
