# Supplementary material for: Fecal Microbial Composition of Ulcerative Colitis and Crohn’s Disease Patients in Remission and Subsequent Exacerbation
Source: PLoS One. 2014 Mar 7;9(3):e90981. doi: 10.1371/journal.pone.0090981 (PMC3946581; doi:10.1371/journal.pone.0090981)
Supplement: Table S5 — Species associations to IBD type in remission state. (DOCX) [file pone.0090981.s010.docx]

Table S5: Species associations to IBD type in remission state

| **Species** | **Effect of IBD type** | **P-value. uncorrected** | **Q-value. FDR** |
| --- | --- | --- | --- |
| ***Oscillospira* undefined species** | UC + | 1.97 * 10^-3^ | 1.46 |
| ***Faecalibacterium* undefined species** | UC + | 2.25 * 10^-2^ | 8.35 |
| **Lachnospiraceae undefined genus** | UC + | 2.76 * 10^-2^ | 4.08 |
| **Lachnospiraceae undefined genus** | UC + | 2.76 * 10^-2^ | 5.11 |
| **Eubacterium rectale** | UC + | 2.76 * 10^-2^ | 6.81 |
